# The Phylogenetic Structure of Reptile, Avian and Uropathogenic *Escherichia coli* with Particular Reference to Extraintestinal Pathotypes

**DOI:** 10.3390/ijms22031192

**Published:** 2021-01-26

**Authors:** Marta Książczyk, Bartłomiej Dudek, Maciej Kuczkowski, Robert O’Hara, Kamila Korzekwa, Anna Wzorek, Agnieszka Korzeniowska-Kowal, Mathew Upton, Adam Junka, Alina Wieliczko, Radosław Ratajszczak, Gabriela Bugla-Płoskońska

**Affiliations:** 1 Department of Microbiology, Institute of Genetics and Microbiology, University of Wrocław, 51-148 Wrocław, Poland; bartlomiej.dudek@uwr.edu.pl (B.D.); kamila.korzekwa@uwr.edu.pl (K.K.); 2 Department of Epizootiology and Clinic of Bird and Exotic Animals, Wrocław University of Environmental and Life Sciences, 50-366 Wrocław, Poland; maciej.kuczkowski@upwr.edu.pl (M.K.); alina.wieliczko@upwr.edu.pl (A.W.); 3 Virology Department, Manchester University NHS Foundation Trust, Manchester M13 9WL, UK; 4 Polish Collection of Microorganisms PCM, Department of Immunology of Infectious Diseases, Hirszfeld Institute of Immunology and Experimental Therapy, Polish Academy of Sciences, 53-114 Wrocław, Poland; anna.wzorek@hirszfeld.pl (A.W.); agnieszka.korzeniowska-kowal@hirszfeld.pl (A.K.-K.); 5 School of Biomedical & Healthcare Sciences, Faculty of Health, University of Plymouth, Plymouth PL4 8AA, UK; mathew.upton@plymouth.ac.uk; 6 Department of Pharmaceutical Microbiology and Parasitology, Medical University of Wrocław, 50-556 Wrocław, Poland; adam.junka@umed.wroc.pl; 7 Zoological Garden in Wrocław, 51-618 Wrocław, Poland; r.ratajszczak@zoo.wroc.pl

**Keywords:** extraintestinal *E. coli*, phylogenetic analysis, virulence genes

## Abstract

The impact of the Gram-negative bacterium *Escherichia coli* (*E. coli)* on the microbiomic and pathogenic phenomena occurring in humans and other warm-blooded animals is relatively well-recognized. At the same time, there are scant data concerning the role of *E. coli* strains in the health and disease of cold-blooded animals. It is presently known that reptiles are common asymptomatic carriers of another human pathogen, *Salmonella*, which, when transferred to humans, may cause a disease referred to as reptile-associated salmonellosis (RAS). We therefore hypothesized that reptiles may also be carriers of specific *E. coli* strains (reptilian *Escherichia coli*, RepEC) which may differ in their genetic composition from the human uropathogenic strain (UPEC) and avian pathogenic *E. coli* (APEC). Therefore, we isolated RepECs (*n* = 24) from reptile feces and compared isolated strains’ pathogenic potentials and phylogenic relations with the aforementioned UPEC (*n* = 24) and APEC (*n* = 24) strains. To this end, we conducted an array of molecular analyses, including determination of the phylogenetic groups of *E. coli*, virulence genotyping, Pulsed-Field Gel Electrophoresis-Restriction Analysis (RA-PFGE) and genetic population structure analysis using Multi-Locus Sequence Typing (MLST). The majority of the tested RepEC strains belonged to nonpathogenic phylogroups, with an important exception of one strain, which belonged to the pathogenic group B2, typical of extraintestinal pathogenic *E. coli*. This strain was part of the globally disseminated ST131 lineage. Unlike RepEC strains and in line with previous studies, a high percentage of UPEC strains belonged to the phylogroup B2, and the percentage distribution of phylogroups among the tested APEC strains was relatively homogenous, with most coming from the following nonpathogenic groups: C, A and B1. The RA-PFGE displayed a high genetic diversity among all the tested *E. coli* groups. In the case of RepEC strains, the frequency of occurrence of virulence genes (VGs) was lower than in the UPEC and APEC strains. The presented study is one of the first attempting to compare the phylogenetic structures of *E. coli* populations isolated from three groups of vertebrates: reptiles, birds and mammals (humans).

## 1. Introduction

The complex phylogenetic structure of *Escherichia coli* species [1,2,3] is strong evidence for the clonal evolution of this bacterium; however, recent publications suggest that recombination processes are playing a greater significant role [4,5,6,7,8]. Four main phylogenetic groups of *E. coli* have been determined. Groups A and B1 mainly include nonpathogenic commensal *E. coli* strains, while extraintestinal pathogenic *E. coli* are clustered to groups B2 and D [8,9,10]. The use of Multi-Locus Sequence Typing (MLST) has enabled a more accurate determination of the phylogenetic structure of *E. coli* species by targeting their housekeeping genes and assigning strains to an appropriate Sequence Type (ST) [9,11,12]. As a result, not only new hybrid phylogroups of *E. coli* have been discovered [13], but it has also been revealed that 80–85% of *E. coli* strains might have been previously assigned to incorrect phylogenetic groups [14,15]. Therefore, new phyllo groups have been formed and referred to as group C, which contains nonpathogenic strains of *E. coli*, closely related to representatives of the B1 group [16], group E, whose representatives (including, among others, *E. coli* O157:H7 strains) are associated with strains of group D [13,14,15] and, finally, group F, clustering extraintestinal pathogenic *E. coli* closely related with the B2 group representatives [13]. In addition to the aforementioned main groups, a phylogenetic unit referred to as the “cryptic clade” has been distinguished, consisting of new species of the genus *Escherichia* [17]. The latest Clermont report revealed a new group intermediate between the F and B2 phylogroups, which has been designated as phylogroup G [18].

Extraintestinal *E. coli* (ExPEC) strains are clustered in the groups B2, E, D and F and characterized by great diversity with regards to the sets of virulence factors, mechanisms of the pathogenesis and types of resulting infections. Therefore, these strains are assigned to various pathotypes. It has been determined that the ExPEC strains isolated from humans and other warm-blooded animals (cats, dogs, pigs, cattle and fowl) are of high genetic similarity, which is believed to be a result of the high plasticity of the *E. coli* genome and of the transmission of *E. coli* between humans and these animals [19,20,21,22]. Due to the wide prevalence and significant role of ExPEC in the pathogenesis, these strains have been the subject of intense scientific investigations [23,24]. At the same time, the knowledge of the virulence traits and genetic compositions of the *E. coli* strains isolated from cold-blooded animals is extremely sparse; only a few reports have been published on the isolation of *E. coli* strains from the intestines of reptiles [25,26].

Increasing overpopulation and environmental pollution lead to an increased exposure of wild-living animals to wastes containing human pathogens, such as *E. coli*. Since reptiles are frequent carriers of Gram-negative pathogenic bacteria, including *Salmonella*, which is able to cause a serious disease called Reptile-Associated Salmonellosis (RAS) [27], we hypothesized that reptiles may also carry other Gram-negative bacteria, such as ExPEC, as their natural microbiota and transmit them [28].

Therefore, the main aim of the present study was to determine the phylogenetic relationships of the RepEC, UPEC and APEC strains and to assess the distribution of virulence genes (VGs) in their genomes.

## 2. Results

Among all bacterial strains isolated from reptile’s feces and assigned to the *E. coli* species using biochemical testing and a Matrix-Assisted Laser Desorption/Ionization Time-of-Flight Mass Spectrometry (MALDI-TOF MS) analysis, (RepEC strains (*n* = 35) include: strains from snakes (*n* = 15), strains from turtles (*n* = 14) and from lizards (*n* = 6)), we selected to further study 24 strains. The datasets analyzed during the current study are available from the Polish Collection of Microorganisms PCM (Wroclaw, Poland) on reasonable request. *E. coli* were identified using MALDI-TOF MS with log scores ranging from 2.205 to 2.878, which indicate highly probable and probable species identification, respectively.

### 2.1. Phylogenetic Groups of E. coli

**Figure 1:**
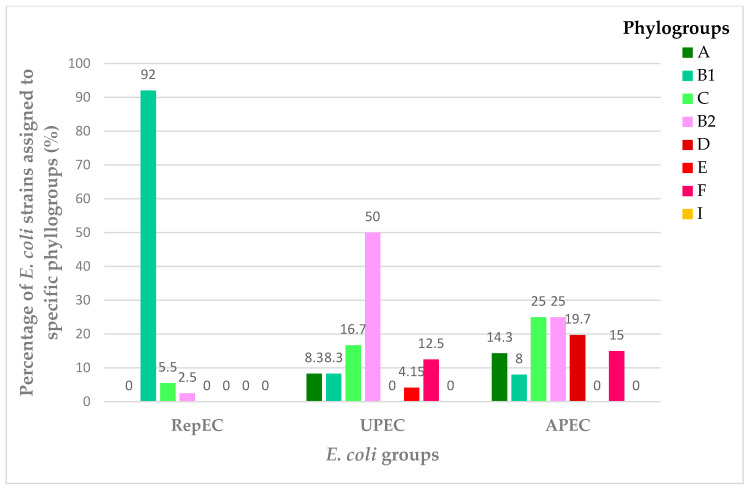
This percentage (%) share of *Escherichia coli* (*n* = 72) strains belonging to specific phylogroups. RepEC—*E. coli* strains isolated from reptiles (*n* = 24), UPEC—uropathogenic *E. coli* strains (*n* = 24) and APEC—avian *pathogenic E. coli* strains causing colibacillosis (*n* = 24). Phylogroups: A and B1 predominantly include nonpathogenic commensal *E. coli* strains, B2 and D include extraintestinal pathogenic *E. coli*, C contains nonpathogenic strains of *E. coli*, closely related with representatives of group B1, E is represented by strains closely related with phylogroup D (including, among others, *E. coli* O157:H7 strains) and F clusters extraintestinal pathogenic *E. coli* closely related with group B2 representatives.

**Figure A1:**
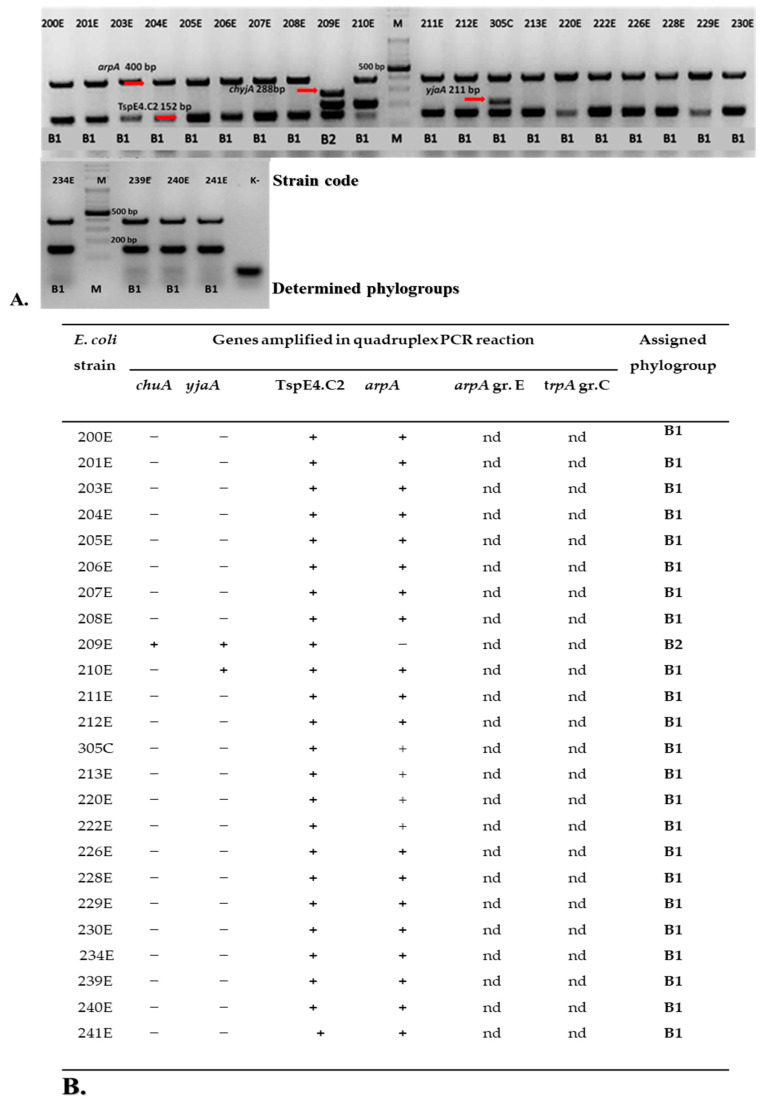
(**A**) Electrophoretograms of amplified products of the quadruplex-PCR method [15,18] with primers targeting Table 288. bp)*, yjaA* (211 bp)*, arpA* (400 bp) and a genetic region with an unknown function: TspE4C2 (152 bp) conducted for the RepEC (*n* = 24)-tested strains. (**B**) Assignation of RepEC (*n* = 24) strains to the phylogroups. M—DNA mass marker.

**Figure A2:**
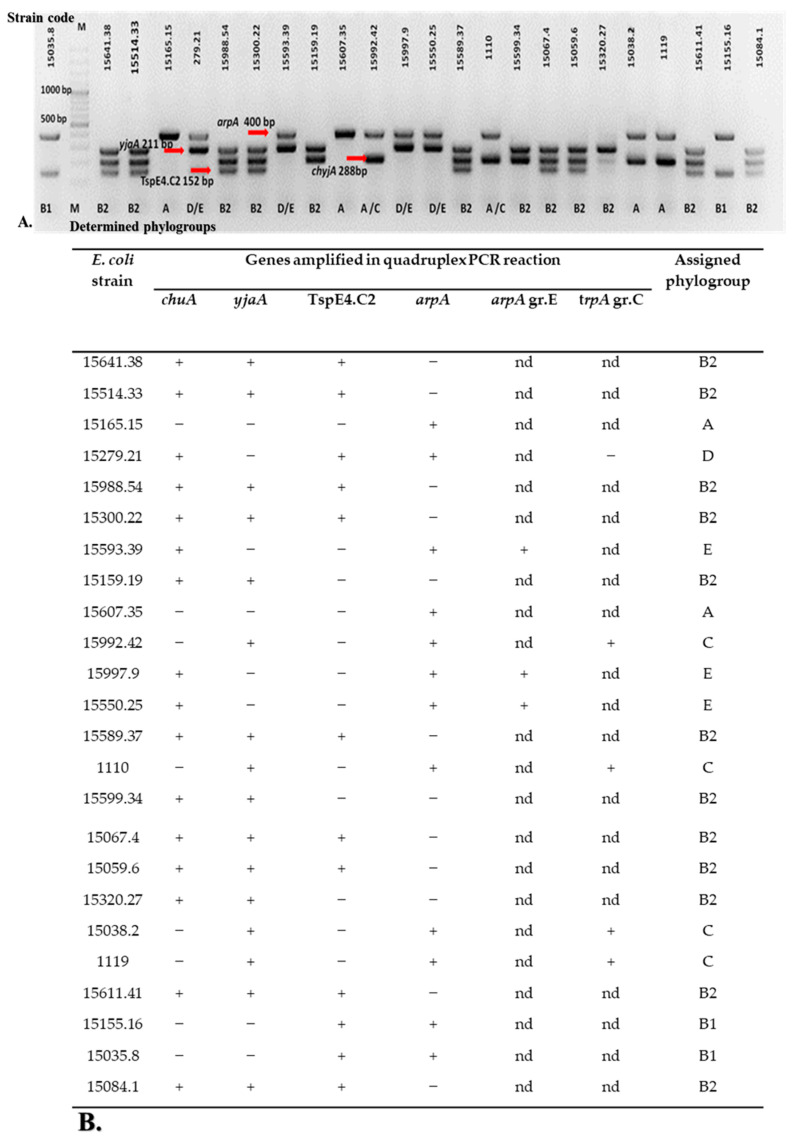
(**A**) Electrophoretograms of amplified products of the quadruplex-PCR method [15,18] with primers targeting the genes: chuA (288 bp), yjaA (211 bp), arpA (400 bp) and a genetic region with an unknown function: TspE4C2 (152 bp) conducted for the UPEC-tested strains (*n* = 24). (**B**) Assignation of UPEC strains (*n* = 24) to the phylogroups. M—DNA mass marker.

**Figure A3:**
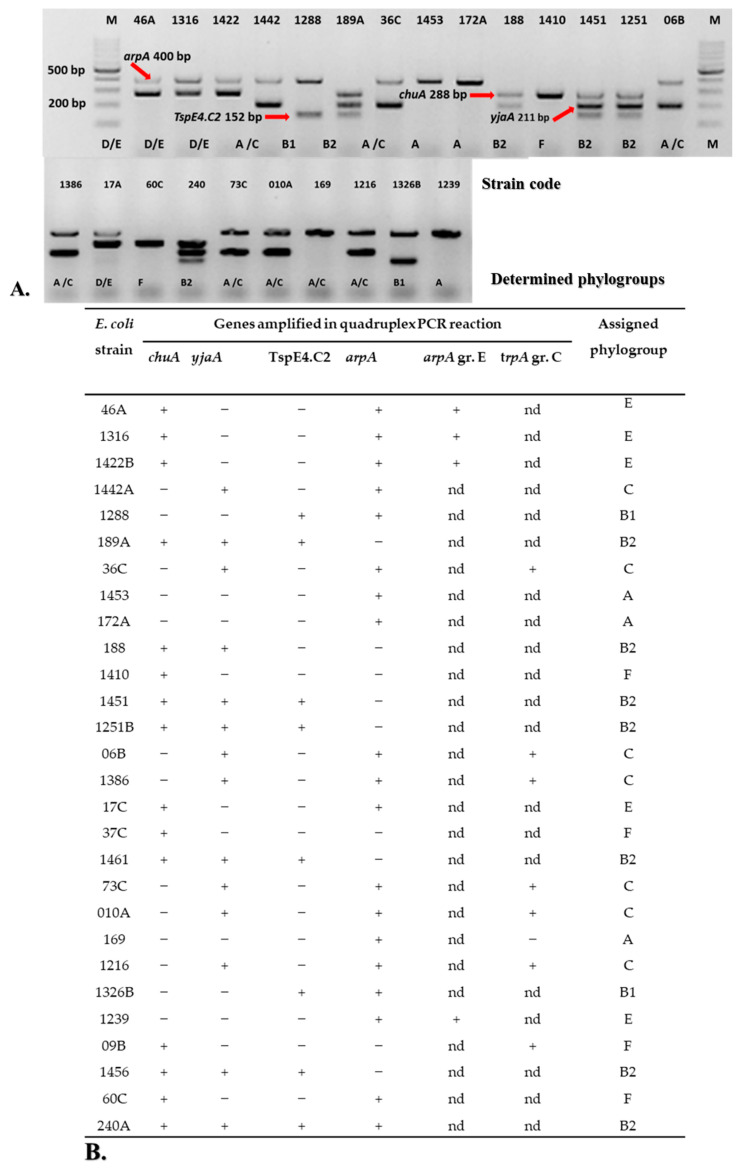
(**A**) Electrophoretograms of amplified products of the quadruplex-PCR method [15,18] with primers targeting the genes: chuA (288 bp), yjaA (211 bp), arpA (400 bp) and a genetic region with an unknown function: TspE4C2 (152 bp) conducted for the APEC-tested strains (*n* = 24). (**B**) Assignation of APEC strains (*n* = 24) to the phylogroup. M—DNA mass marker.

The assignment of the RepEC, UPEC and APEC strains to the respective phylogroups is presented in Figure 1 and Figure A1, Figure A2 and Figure A3 (Appendix A). The results presented in Figure 1 and Figure A1 indicate that 92% and 5% of RepEC strains belonged to the nonpathogenic B1 and C groups, respectively. It should be noted here that one RepEC strain, namely 209E, belonged to the pathogenic B2 phylogroup.

A high percentage of UPEC strains belonged to the B2 phylogroup (50%) (*n* = 12). The percentage shares of specific UPEC strains within the remaining commensal phylogroups were as follows: 17% of UPEC strains (*n* = 4) belonged to Group C, 13% (*n* = 3) were Group E, 8 % (*n* = 2) belonged to group A, 8 % (*n* = 2) belonged to group B1 and 4% (*n* = 1) were from Group D (Figure 1 and Figure A2). The percentage distribution of the phylogroups among the tested APEC strains was relatively homogenous (Figure 1 and Figure A3). They belonged to the following nonpathogenic groups: C (25%, *n* = 7), B2 (25%, *n* = 7), E (18%, *n* = 5), F (14%, *n* = 4), A (11%, *n* = 3) and B1 (7%, *n* = 2).

### 2.2. Virulence Gene Typing

**Figure 2:**
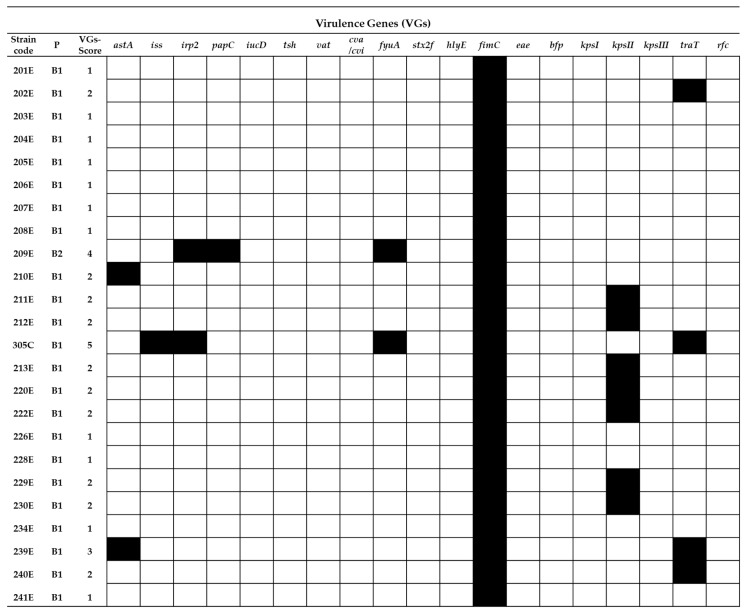
Virulence gene (19 VG) profiles of the tested RepEC strains (*n* = 24). P—phylogenetic group, 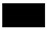 = presence of the gene and 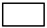 = absence of the gene.

Figure 2 presents a map containing VGs profiles of specific RepEC strains. The VG score for the RepEC strains ranged from 0 (strain 148E and 152E) to 5 (strain 305C). Except for the *fimC* gene (the prevalence of *fimC* for RepEC equaled 100%), the frequency of other tested VGs among RepEC was low or very low. The frequency of VGs detected in RepEC was as follows: *astA* (8%), *iss* (4%), *irp2* (8%), *papC* (4%), *fyuA* (8%), *kpsII* (29%) and *traT* (16%). Other VGs targeted were not detected among the RepEC strains.

**Figure 3:**
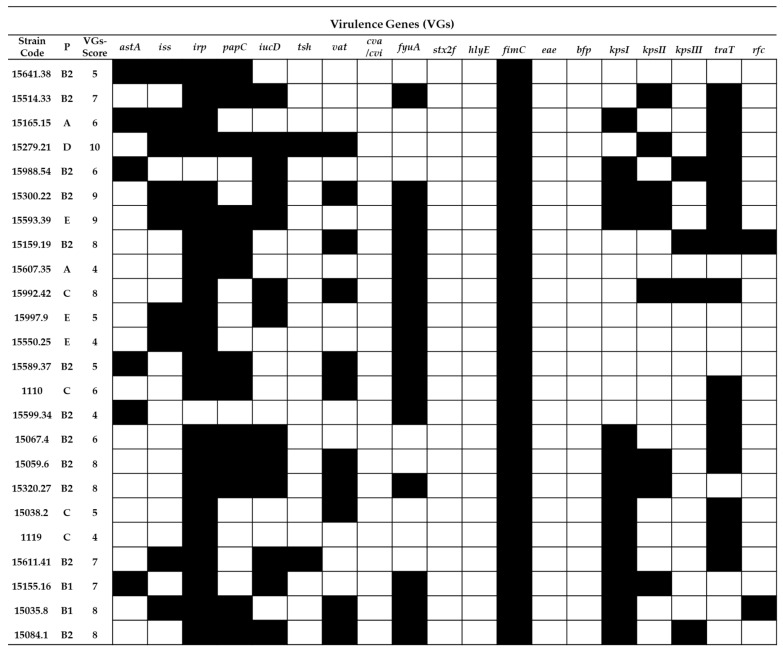
Virulence gene (19 VG) profiles of the tested UPEC strains (*n* = 24). P—phylogenetic group, 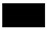 = presence of the gene and 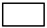 = absence of the gene.

The analyzed UPEC strains (Figure 3) displayed a high prevalence of VGs: *irp2* 92%, *iucD* 54%, *fuyA* 62.5%, *papC* 54%, *traT* 62.5% and *kpsI* 54%. The VG score for the UPEC strains ranged from 4 (strains 15607.35, 15550.25, 15599.34 and 1119) to 9 (strains 15279.21, 15593.39 and 15300.22). The presence of the following VGs were not detected among the tested UPEC strains: *stx2f, hlyE, eae* and *bfp.*

**Figure 4:**
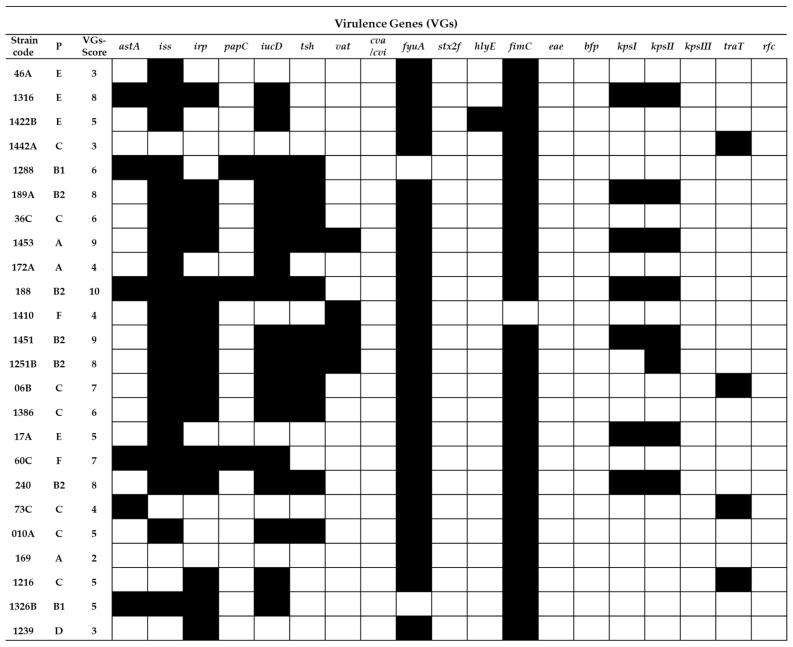
Virulence gene (19 VG) profiles of the tested APEC strains (*n* = 24). P—phylogenetic group, 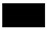 = presence of the gene and 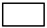 = absence of the gene.

The APEC strains (Figure 4) were characterized by high and very high prevalence of the following VGs: *iss* 76%, *fyuA* 92%, *irp2* 62.5%, *iucD* 70%, *kpsI* 29% and *kpsII* 33%. The VG score values determined for APEC strains ranged from 2 (strain 169) to 10 (strain 188). The presence of the following VGs were not detected among the tested APEC strains: *cva/cvi, stx2f*, *eae*, *bfp* and *kpsIII*.

**Figure 5:**
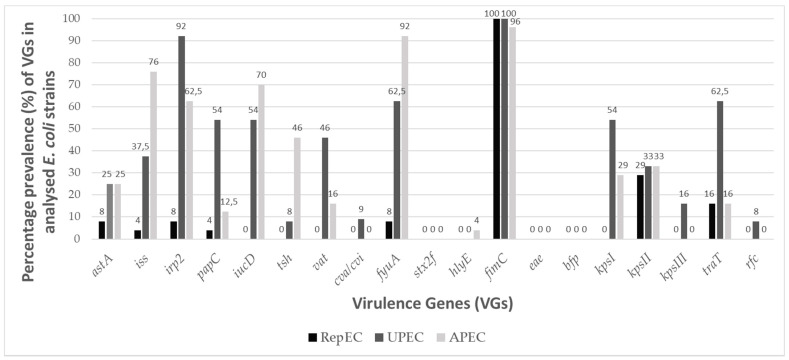
The percentage prevalence of VGs among the RepEC (*n* = 24), UPEC (*n* = 24) and APEC strains (*n* = 24).

In general, the frequency of occurrence of VGs in the RepEC strains was lower than in the UPEC and APEC strains (Figure 5). Only two noticeable exceptions were observed. One concerned the prevalence of the *fimC* gene, which was detected in 100% of the RepEC and UPEC isolates and 96% of the APEC isolates, while the other concerned the prevalence of the *kpsII* gene, whose frequency of occurrence was comparable between RepEC, UPEC and APEC (29%, 33% and 33%, respectively).

The following *E. coli* strains were selected for further analysis with the use of Pulsed-Field Gel Electrophoresis-Restriction Analysis (RAE-PFGE) and MLST: UPEC 15279.21, 1119 and 15035.8; APEC1288, 1239, 189A and 60C and RepEC 200E, 209E, 212E and 305C. The rationale behind this choice was the prevalence of one of the following VGs: *iss*, *tratT*, *kpsII* and *rfc.*

### 2.3. Analysis of Phylogenetic Relationships between E. coli Strains from Different Hosts

**Figure 6:**
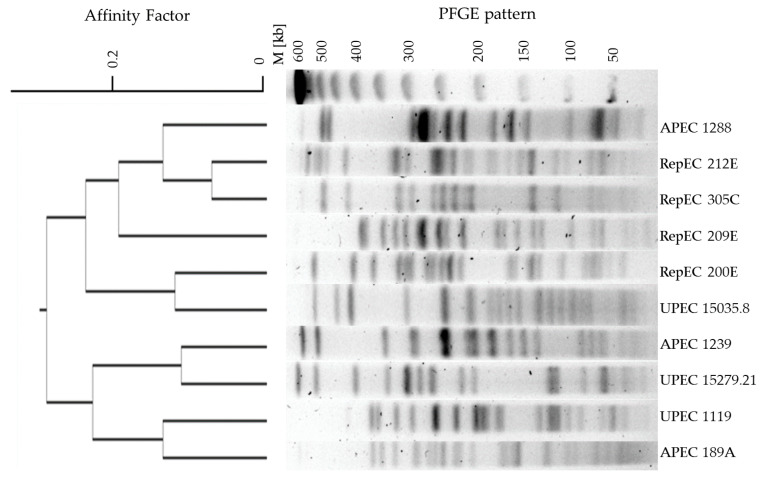
Dendrogram displaying the relationships between the selected selected *E. coli* strains (*n* = 10), including RepEC (*n* = 4), UPEC (*n* = 3) and APEC (*n* = 3), based on the results of the Pulsed-Field Gel Electrophoresis-Restriction Analysis (RAE-PFGE). The dendrogram was created using the Unweighted Pair Group Method with Arithmetic Mean (UPGMA) method. (http://insilico.ehu.es/dice_upgma/index.php). M—molecular weight marker.

The Pulsed-Field Gel Electrophoresis with Restriction Analysis (RAE-PFGE) was conducted, resulting in restriction profiles of 41 DNA fragments displaying high diversity (Figure 6). Each of the tested *E. coli* strains had a unique restriction profile that differed by at least one DNA fragment from the other strains. Additionally, strain 60C (belonging to the APEC pathogroup) appeared to be nontypeable. 

### 2.4. Multi-Locus Sequence Typing

The assignment of *E. coli* strains to specific STs was performed by means of Multi-Locus Sequence Typing (MLST). The results of the MLST analysis and detailed descriptions of the STs assigned to the tested *E. coli* strains are presented in Table 1.

## 3. Discussion

*Escherichia coli* species display a high genetic diversity, genome plasticity and adaptability to different ecological niches. *E. coli* are part of the microbiota of warm-blooded animals and may act as pathogens causing intestinal or extraintestinal infections posing a serious threat to health and survival [38,39].

Pathogenic *E. coli* strains, including extraintestinal pathogenic *Escherichia coli* (ExPEC), have different virulence genes and different mechanisms of pathogenesis, causing a broad range of diseases. The ExPEC include UPEC, Neonatal Meningitidis *E. coli* (NMEC), Sepsis Causing *E. coli* (SEPEC) and APEC [39]. Due to their high capacity for adaptation, ExPEC strains may spread and thrive in different environments. From an ecological and epidemiological perspective, it is of paramount importance to establish the possible routes of contamination by these bacteria in humans and other species (both domesticated and wild-living). A genetic analysis is one of the most important tools serving this purpose. Warm-blooded animals (cats, dogs, cattle, pigs and horses) are already-known reservoirs of ExPEC strains [39,40], but knowledge about the occurrence of *E. coli* in cold-blooded animals is extremely scant. It should be noted that cold-blooded reptiles are frequent asymptomatic carriers of *Salmonella*, a Gram-negative microbe closely related to *E. coli. Salmonella* that may cause a severe disease called Reptile-Associated Salmonellosis [41]. In consideration of the above-presented facts, we hypothesized that *E. coli* may also inhabit the digestive tract of reptiles. Our aim was not only to fill the gap in the knowledge of the composition of intestinal microbes in reptiles but, most of all, to determine whether these cold-blooded animals may be a source of ExPEC pathotypes, which are widely disseminated in the natural environment. In order to address these issues, we conducted a thorough genetic analysis of *E. coli* strains isolated from reptiles (RepEC) and compared findings with the results of an analysis of two well-known pathotypes of *E. coli*, namely UPEC and APEC.

According to the authors’ best knowledge, there are very few reports on the isolation of *E. coli* from reptiles [25,42]. In 2017, 142 *E. coli* strains were isolated from 60 exotic reptiles seized during an airport customs inspection in Frankfurt [43]. In Brazil, nine strains of *E. coli* were collected from 78 lizards [44]; screening tests of 447 wild-live reptiles resulted in the isolation of 45 *E. coli* strains in Austria [32], whereas, in Germany, two *E. coli* isolates were detected in 56 domesticated and wild-living reptiles [42]. Two other screening exercises conducted in Australia [45] and in Cuba [26] confirmed that the prevalence of *E. coli* in the intestinal microbiome is significantly lower in cold-blooded animals compared with warm-blooded ones. In the majority of the previous studies [26,45], no attempts were made to characterize the *E. coli* isolates at the genetic level. In our study, we isolated 24 *E. coli* strains from 120 feces samples collected from reptiles belonging to the collection of the Zoological Garden in Wrocław. Therefore, our data are consistent with the above-mentioned reports [25,26], and they indicate that the prevalence of *E. coli* in the microbiota of reptiles may be considered to be low. The prevalence of *E. coli* in reptiles in Poland has not been reported previously. *E. coli* strains isolated from reptiles may display phenotypic traits typical of pathogenic *E. coli* strains. The strains isolated from reptile feces in Cuba had highly mucoid colonies and were hemolytic when grown on agar containing ovine blood 26]. This observation is consistent with our previous findings in the identification procedure of *E. coli* with ambiguous phenotypes, isolated from the feces of a Bell’s Hinge-back Tortoise (*Kinixys belliana*) [46]. The isolated RepEC strain, referred to as strain 305C in the current study, had a mucoid colony morphology and a thick cell capsule, characteristic of the genus *Klebsiella*. Using the MALDI-TOF MS analysis, the strain referred to above was assigned to the species *Citrobacter braakii*; however, biochemical tests indicated that it belongs to the *E. coli* species. The conclusive results were finally obtained with the use of 16S rRNA gene sequencing and confirmed that the strain was a member of the *E. coli* species [46], and further studies revealed this 305C RepEC strain may belong not to the previously described Sequence Type but belong to the ST88 clonal complex. ST88 includes ExPEC strains (UPEC, APEC and SEPEC) [29]. Therefore, determination of the 305C RepEC strain to any known ST confirms its unique and atypical traits. As previously mentioned, our aim was to characterize RepEC strains with genetic analysis tools by comparing three *E. coli* groups from different sources. The present study is the first to communicate the phylogenetic comparison of *E. coli* strains isolated from three classes of vertebrates, i.e., reptiles, mammals and birds. In the first experimental setting, we determined the phylogroups for the RepEC strains (Figure 1 and Figure A1, Figure A2 and Figure A3). The majority (95%) of them were assigned to the nonpathogenic phylogroup B1. The results obtained are consistent with those presented by Gordon and colleagues, who tested the prevalence of *Enterobacteriaceae* among mammals, birds and cold-blooded animals in Australia [45]. In the case of cold-blooded animals, *E. coli* belonging to the phylogroup B2 were very rarely isolated [45]. In our study, one RepEC strain 209E was assigned to the phylogroup B2, typical of ExPEC strains. A majority of the UPEC and APEC strains were assigned to the group B2 (50% and 25%, respectively). Only 8.3% and 7% of the UPEC and APEC strains, respectively, were assigned to the group B1.

There are no reports describing the prevalence of VGs in RepEC strains in the global scientific literature. In our study, we determined that RepEC strains show a low prevalence of VGs (VG score: 0–5) in comparison with the tested UPEC (VG score: 4–9) and APEC strains (VG score: 2–10). One of the two noticeable exceptions was the gene *fimC* present in 96% of the RepEC strains. This may be explained by the fact that this gene is essential for the production of type I fimbriae, a universal virulence factor [47] ubiquitous within the species. The foregoing assumption may be additionally confirmed by the fact that, in our study, the *fimC* gene was also detected in 100% and 98% of the tested UPEC and APEC strains, respectively. The main function of type 1 fimbriae in *E. coli* pathogenesis is adhesion to the host tissue, especially in urinary tract infections (UTIs) [48,49,50]. Type 1 fimbriae help pathogenic bacteria to avoid host immune defenses (protection against phagocytosis) [51]. Expression of the *fimC* gene enhances the virulence of *E. coli*, while deletion of this virulence factor could result in the loss of pathogenicity [52]. However, it has been shown that a low-level expression of the *fimC* gene in commensal *E. coli* confers nonpathogenic functions [47]. The low-level expression of *fimC* in commensal strains could enhance their ability to persist in the digestive tract of the host by enabling avoidance of the immune defense activity by *E. coli* cells and maintain balance with the host immune system. Additionally, the prevalence of the *kpsII* gene (encoding capsular antigen) was comparable between the tested strains. The *kpsII* gene encodes extracellular capsules and plays a crucial role in bacterial resistance to the host immune system [53]. The prevalence of other VGs among the RepEC strains was very low in comparison with the APEC and UPEC strains. However, the other VGs detected in RepEC strains were typical of ExPEC pathotypes. The following VGs were identified among RepEC isolates: *iss* and *traT*–genes responsible for an increased survival in the serum, *papC*—encoding a subunit of P fimbriae, one of the most important virulence factors in urinary infections, *iucD* and *fyuA*—genes encoding siderophore systems and *kpsI* and *kpsII*—genes encoding extracellular capsules [27,54]. On the other hand, although ExPECs are pathogens, they are also a part of the normal gut microbiome, where they live as commensals, and their VG expressions are silenced [54]. As mentioned for virotyping among RepEC strains, the presence of *iucD, tsh, vat, cva/cvi, stx2f, hlyE, eae, bpf, kpsIII* and *rfc* were not observed. These genes encode a number of functions (Table A3) [27,54]. The first seven of the aforementioned genes are connected with the pathogenesis of E. coli causing intestinal infections. As RepEC strains do not have those VGs, they may be considered as not being intestinal pathogens. The RepEC strain 209E was assigned to the phylogroup B2 (typical of pathogenic strains) with the VG score of 4. We detected five virulence genes in the genome of the RepEC strain 305C (with ambiguous phenotypes). Based on the classification presented by Russo and Johnson [27], the RepEC strains 209E and 305C could be considered as potentially pathogenic ExPEC. Thirty percent of the tested RepEC isolates had the following genes: *iss* and *traT* (increased serum survival), *fimC* (type 1 fimbriae) and *irp2* and *fyfA* (siderophores). The above set of VGs is typical of extraintestinal pathogens supporting survival in the host organism and resistance to the host immune system.

With the use of MLST (Table 1), we determined the ST of the selected RepEC strains (200E, 209E, 212E and 305C). They were typical of pathogenic *E. coli* strains. Strain 200E belongs to ST446 [41,42], which mainly includes the APEC strains and other ExPEC pathotypes, which are pathogenic for humans. The RepEC strain 209E was assigned to ST681, typical of the ExPEC strains, pathogenic for animals and humans [29,37]. Strain 212E belongs to an ST characteristic for UPEC and other extraintestinal pathogens [29,55]. The RepEC strain 305C was not assigned to any ST that was described before. In the literature, there is only one study available in which *E. coli* isolated from cold-blooded animals was analyzed using MLST [43]. One of the two *E. coli* strains with a multidrug resistant (MDR) phenotype isolated from reptiles in Taiwan was assigned to ST117, which includes a heterogenous group of ExPEC pathogens, while the other was assigned to an ST typical of nonpathogenic *E. coli* strains [43]. ST117 and ST88 have been previously reported in colistin-resistant mcr-1-positive pathogenic “zoonotic-related” *E. coli* [56,57].

The RAE-PFGE analysis revealed a high level of diversity in the tested *E. coli* strains (Figure 6). The dendrogram created showed that some of the tested RepEC strains cluster with the tested UPEC and APEC strains. The dendrogram created with the use of the RAE-PFGE analysis showed that strains: 15035.8 (UPEC) and 200E (RepEC) share some genetic material. These results may indicate that these pairs of strains share a some of genetic material and exhibit close phylogenetic relationships. All tested with RAE-PFGE strains isolated from reptiles have various genetic patterns. Strains 200E, 209E and 212E were isolated from snakes, but there are no closer genetic relationships. However, the 212E strain isolated from snakes and 305C strain isolated from turtles showed some similarities in their genetic profiles (Figure 6).

Based on these results, it is difficult to establish whether *E. coli* isolated from reptiles could have been derived from extraintestinal human or avian pathogenic *E. coli* strains during the evolutionary process or if they have been directly transferred to reptiles in the process of contamination via direct contact with wastewater. In light of the obtained results, it is clear that *E. coli* isolated from reptiles is an important target of scientific research. Developed in the system of cold-blooded animals and exposed to the activity of the reptilian immune system, RepEC may develop unique virulence traits that could pose a threat to the health of other animals and humans following any reptile–human and reptile–animal contact. The study presented here makes a contribution to the data of the genetic characteristics of RepEC strains and provides a meaningful insight that could be useful in the acquisition of knowledge of the possible routes of pathogen transmission and subsequent infections. Reptile breeders and people keeping cold-blooded animals as domestic pets should maintain a high level of personal hygiene and be aware of the potential health risk during contact with these animals.

## 4. Materials and Methods

### 4.1. Bacterial Strains

In the presented research, strains of *E. coli* (*n* = 72) were used belonging to the following groups. *E. coli* isolated from feces of healthy reptiles, which we referred to as Reptile-associated *E. coli*, RepEC (*n* = 24). Uropathogenic *E. coli*, UPEC (*n* = 24), isolated in Lower Silesia from urine samples (Dialab Medical Laboratory, Wrocław, Poland). The RepEC and UPEC strains are part of the collection of the Department of Microbiology of University of Wrocław, Poland (Table A1). *E. coli* causing systemic infections among birds (colibacillosis), referred to as Avian Pathogenic *E. coli* (APEC) (*n* = 24). APEC strains used in this study belong to the collection of the Department of Epizootiology and Clinic of Bird and Exotic Animals of Wrocław University of Environmental and Life Sciences, Wrocław, Poland. APEC strains were isolated from extraintestinal organs of poultry exhibiting colibacillosis, such as the liver or heart (Table A1).

We conducted a complete isolation and identification procedure for *E. coli* strains isolated from feces samples from reptiles. Samples of reptile feces or cloaca’s swabs were collected by qualified Zoo’s workers of the Zoological Garden in Wrocław, Poland. We obtained samples (*n* = 103) in transport media; after that, all samples were transferred onto Brain Heart Infusion (BHI) broth preincubated in 37 ˚C and cultured on the mentioned below selective media. Since the contamination of samples, especially collected from reptile feces, could be the source of bias for the results of the subsequent phylogenetic analyses, the samples were cultured on appropriate, selective media: MacConkey Agar, Xylose Lysine Deoxycholate (XLD) Agar and Endo Agar. Moreover, we identified bacterial strains and confirmed that they are pure cultures with the application of the MALDI-TOF MS method.

Uropathogenic *E. coli* strains, UPEC (*n* = 24) strains, were obtained to study with scientific cooperation from the Dialab Medical Laboratory, Wrocław, Poland. Uropathogenic *E. coli strains* (UPEC strains) were identified according to the recommendations of the Centers for Disease Control and Prevention (CDC), Expert Committee on Biological Standardization World Health Organization (WHO)-Basic Laboratory Procedures in Clinical Bacteriology and the recommendations of the National Medical Microbiology Advisor and the National Center for Drug Susceptibility [58,59,60,61].

Whereas APEC strains (*n* = 24) isolated from extraintestinal organs (heart and liver) of poultry with colibacillosis were obtained to study with scientific cooperation from the Department of Epizootiology and Clinic of Bird and Exotic Animals of Wrocław University of Environmental and Life Sciences.

We also confirmed the purity of UPEC and APEC with diagnostic culture media: MacConkey Agar, XLD Agar and Endo Agar. All tested RepEC, UPEC and APEC strains were isolated and collected in Lower Silesia in the years 2010–2014 and are listed in Table A1 (Appendix A).

### 4.2. Isolation and Identification of Bacterial Strains

Isolates were identified using traditional biochemical testing and mass spectrometry [37]. Bacterial strains used in this study were identified as *E. coli* species using Bruker MALDI-TOF MS systems. All analyses were performed with the AUTOFLEX III SmartBeam (Bruker Daltonics, Bremen, Germany) and UltraflExtreme (Bruker Daltonics, Bremen, Germany) using the Biotyper 3.0 software consisting of a database containing 4613 entries. Identification by MALDI-TOF MS was confirmed by multiple tests. Identification of isolates at species level with log scores ≥ 2.300 was considered highly probable. Log scores > 2.0 indicated a high probability of identification at the species level. Log scores between 1.700–1.999 yielded probable identification at the genus level. Scores < 1.800 were interpreted as unreliable identification.

### 4.3. DNA Extraction

Whole-genome DNA were extracted from all tested bacterial strains (*n* = 72) with the application of a commercial versatile kit for genomic DNA purification (Genomic Mini, AA-Biotechnology, Gdynia, Poland) and stored in −20 °C.

### 4.4. Phylogenetic Groups of E. coli

In order to assess the genetic diversity, phylogenetic groups of the tested *E. coli* strains were determined by a quadruplex-PCR method [23], with primers targeting the genes *chuA* (288 bp), *yjaA* (211 bp), *arpA* (400 bp) and a genetic region with an unknown function, *TspE4C2* (152 bp). In the case of nonconclusive results, for strains belonging to groups D/E or A/C, the PCR reaction was performed to detect the *arpA* (301 bp) and *trpA* (219 bp) genes.

For the reaction, there was set up 25 µl of PCR mixture per sample containing 50 ng of template DNA, 2-U Taq polymerase DNA DreamTaq™ Green (Thermo Scientific, Vilnius, Lithuania), 2.5 µL of 10 × DNA DreamTaq™ Green Buffer (Thermo Scientific, Vilnius, Lithuania), 200-mM dNTP (Thermo Scientific, Vilnius, Lithuania) and 20 pmol each of the primers (Genomed, Warszawa, Poland). PCR amplifications were performed with parameters as follows: 95 °C for 4 min and 30 cycles of denaturation (30 s, 95 °C), annealing (20 s, 59 °C for quadruplex and phylogroup C and 20 s, 57 °C for phylogroup E), extension steps (1 min, 72 °C) and final extension (10 min, 68 °C). The list of the primers (Genomed, Warszawa, Poland) used for the determination of the phylogenetic groups of *E. coli* is presented in Table A2 (Appendix A).

### 4.5. Virulence Genotyping

All *E. coli* strains were tested for 19 VGs, including those related to the pathogenicity of diarrhoeagenic E. coli (*astA, stx2, eae* and *bfp*); pathogenicity of APEC (*iss, irp2, iucD, tsh, vat, fyuA, hlyE, fimC* and *cvi/cva*) and genes characteristic for UPEC (*papC, kpsMTII, kpsMT (K1), kpsMTI, rfc* and *traT*) (see Table A3 for more details). These 19 VGs were targeted by three multiplex-PCR reactions according to the literature [38,62,63]. The list of primers used in the determination of VGs in *E. coli* (Genomed, Warszawa, Poland) is presented in Table A3 (Appendix A).

On the basis of the PCR results obtained, the percentage values for VG prevalence were assigned to the categories of very low, low, medium, high or very high frequencies, as presented in Table 2.

### 4.6. Gel Electrophoresis of PCR Amplification Products

All PCRs reactions were conducted in a DNA Thermal Cycler T100™ (Bio-Rad, Dublin, Ireland). Amplified products were resolved on a 2% agarose gel (Sigma-Aldrich, Wien, Switzerland) and visualized using Midori Green DNA (Nippon Genetics, Dueren, Germany). Band patterns were visualized under UV light and photographed using a Gel Doc camera system (Bio-Rad) and analyzed with Quantity One software (Bio-Rad). For each PCR reaction, 1 µl of sterile water added to the PCR mixture used as a negative control in the experiment. PCR assays were performed twice to ensure that the strains were correctly assigned to their respective patterns.

For the purpose of the next step of the phylogenetic analyses (RAE-PFGE analysis and MLST), eleven of the tested *E. coli* strains were selected: UPEC 15279.21, 1119 and 15035.8; APEC 1288, 1239, 189A and 60C and RepEC 200E, 209E, 212E and 305C. These strains were selected based on the obtained results of phylogroup assignment and virotyping.

### 4.7. Restriction Analysis Combined with Pulsed-Field Gel Electrophoresis (RAE-PFGE)

The above-mentioned eleven *E. coli* strains were fingerprinted by using the RAE-PFGE method, as per the PulseNet protocol developed by the US Centers for Diseases Control and Prevention [64] (PulseNet). Total DNA was prepared for restriction analysis with the application of the XbaI enzyme (Thermo Fisher Scientific, Vilnius, Lithuania). DNA separation was performed with CHEF DR III (Bio-Rad) under the following conditions: 1% agarose gel (Prona Agarose) in 0.5-M Tris–Borate–EDTA buffer at 14 °C for 19 h at 6.0 V/cm (200 V). Pulse time ranged between 2.2 and 63.8 s. Molecular weight marker ProMega-Markers^®^ Lambda Ladders was used for analysis (Promega, Madison, USA). The gels were stained and visualized as per the PCR reactions above. RAE-PFGE patterns were analyzed by visual assessment, and the dendrograms were generated with the UPGMA method using online software available at http://insilico.ehu.es/.

### 4.8. Genetic Population Structure Analysis of E. coli Strains by Multi-Locus Sequence Typing

MLST analysis of the *E. coli* strains selected (*n* = 11) previously was carried out by using the MLST scheme for *E. coli* developed by Achtman et al. [12,29]. Housekeeping genes, their biological functions and the sequences of the applied primers are presented in Table A4 (Appendix A). Presence of the correct size PCR product was confirmed by agarose gel electrophoresis, as above, using an appropriate DNA marker (100-bp ladder; Sigma-Aldrich). DNA samples were prepared and sequenced by LGC Standards (Manchester, UK), and the obtained sequences were analyzed with CLC Genomics Workbench. The determined allelotypes of the tested *E. coli* strains were assigned to the relevant Sequence Type (ST) according to the http://enterobase.warwick.ac.uk/species/index/ecoli database.

## Figures and Tables

**Table 1:** Results of the assignment of selected *Escherichia coli* strains (*n* = 11), including the reptilian *E. coli* (RepEC) (*n* = 4), uropathogenic strain (UPEC) (*n* = 3) and avian pathogenic strain (APEC) (*n* = 4), to the Sequence Type on the basis of their allelic profiles of their house-keeping genes (allelotypes) and descriptions of the STs according to the Warwick University Multi-Locus Sequence Typing (MLST) database. P—phylogroup.

Group of *E. coli*	*E. coli* Strain Code	P	Asigned Sequence Type	Description of STs	Citations
**UPEC** (*n* = 3)	15279.21	D	ST321	ST321 is characteristic of nonpathogenic *E. coli* strains isolated from wild animals.	[29]
	1119	C	ST410	which includes pathogenic *E. coli* strains (human UPEC strains, predominantly).	[29,30]
	15035.8	B1	ST12	ST12 is typical of extraintestinal pathogenic *E. coli* strains causing systemic infections in warm-blooded animals and also typical of *E. coli* causing urinary tract infections (UTIs) among humans.	[29,31]
**APEC** (*n* = 4)	1288	B1	ST1582	ST1582 is characteristic for UPEC strains causing UTIs in horses.	[29]
	1239	D	ST665	ST665 is typical of nonpathogenic *E. coli* strains isolated from poultry.	[29,32]
	189A	B2	ST131	ST131 is a globally disseminated clone that poses a substantial health risk to humans, causing acute extraintestinal infections, such as serious UTI, urosepsis and sepsis.	[8,29,33,34]
	60C		ST117	ST117, which is a heterogenic group of extraintestinal pathogenic *E. coli* strains.	[29,35]
**REPEC** (*n* = 4)	200E	B1	ST446	ST446 is typical of APEC strains and also of other human pathogenic extraintestinal *E. coli*.	[29,36]
	209E	B2	ST681	ST 681 includes *E. coli* strains pathogenic for humans and other animals.	[29,37]
	212E	B1	ST212	This heterogenous ST mainly includes UPEC strains as well as other pathogenic and nonpathogenic *E. coli* strains.	[29,38]
	305C	B1	New ST!	Strain 305C could not be assigned to any known ST, although it could be a member of the ST88 clonal complex (with differences in two alleles *adk* and *mdh*; unconfirmed data).	-

**Table 2:** Frequency of virulence genes (VGs) occurrence scale.

Percentage Prevalence of VGs	Frequency of VGs
0–10%	Very low
11–20%	Low
21–45%	Medium
46–80%	High
81–100%	Very high

## Data Availability

The data presented in this study are available on request from the corresponding author. The data are not publicly available.

## Appendix A

**Table A1:** The list of *Escherichia coli* strains: RepEC (*n* = 24), UPEC (*n* = 24) and APEC (*n* = 24) used in the study.

Group of *Escherichia coli* Strains					
RepEC		UPEC		APEC	
Strain Code	Source of Isolated Bacteria	Strain Code	Source of Isolated Bacteria	Strain Code	Source of Isolated Bacteria
	*Escherichia coli* strains isolated from feces of reptiles (snakes, lizards and turtles)		*Escherichia coli* isolated from urine samples of patients with symptomatic and asymptomatic urinary tract infections		*Escherichia coli* strains isolated from extraintestinal organs (heart, liver) of poultry with colibacillosis
200E	Snake (*Lamprophis fuliginosus*)	15641.38		46A	
201E	Snake (*Orthriophis taeniurus*)	15514.33		1316	
203E	Lizard (*Pogona vitticeps*)	15165.15		1422B	
204E	Lizard (*Chamaeleo calyptratus*)	15279.21		1442A	
205E	Lizard (*Anolis barbatus*)	15988.54		1288	
206E	Snake (*Epicrates cenchria*)	15300.22		189A	
207E	Snake (*Lamprophis fuliginosus*)	15593.39		36C	
208E	Snake (*Lamprophis fuliginosus*)	15159.19		1453	
209E*	Snake (*Lamprophis fuliginosus*)	15607.35		172A	
210E	Snake (*Lampropeltisgetula*)	15992.42		188	
211E	Snake (*Elaphe schrencki*)	15997.9		1410	
212E	Snake (*Coelognathus radiata*)	15550.25		1451	
305C	Turtle (*Kinixys belliana*)	15589.37		1251B	
213E	Turtle (*Chelonoidis nigra*)	1110		06B	
220E	Turtle (*Geochelone platynota*)	15599.34		1386C	
222E	Turtle (*Testudo marginata*)	15067.4		17A	
226E	Lizard (*Chamaeleo calyptratus*)	15059.6		60C	
228E	Turtle (*Geochelone platynota*)	15320.27		240	
229E	Turtle (*Astrochelysradiata*)	15038.2		73C	
230E	Turtle (*Centrochelys sulcata*)	1119		010A	
234E	Turtle (*Testudo horsfieldii*)	15611.41		169	
239E	Snake (*Lampropeltis triangulum*)	15155.16		1216	
240E	Lizard (*Anolis baracoae*)	15035.8		1326B	
241E	Turtle (*Chelonoidisnigra*)	15084.1		1239	

**Table A2:** The list of the primers (Genomed, Gdynia, Poland) used for the determination of the phylogenetic groups of *E. coli* in the quadruplex-PCR method [15,18].

Gene	Sequences of Primers	PCR Product Size (bp)	Gene Bank ID
*yjaA*	5′-CAAACGTGAAGTGTCAGGAG-3′ 5′-AATGCGTTCCTCAACCTGTG-3′	288	948515
*chuA*	5′-CAAACGTGAAGTGTCAGGAG-3′ 5′-AATGCGTTCCTCAACCTGTG-3′	211	7155958
*TspE4C2*	5′-CACTATTCGTAAGGTCATCC-3′ 5′-AGTTTATCGCTGCGGGTCGC-3′	152	EU240725.1
*arpA*	5′-AACGCTATTCGCCAGCTTGC-3′ 5′-TCTCCCCATACCGTACGCTA-3′	400	7155679
*arpA*	5′-GATTCCATCTTGTCAAAATATGCC-3′ 5′-GAAAAGAAAAAGAATTCCCAAGAG-3′	301	944933
*trpA*	5′-AGTTTTATGCCCAGTGCGAG-3′ 5′-TCTGCGCCGGTCACGCCC-3′	219	912862
*trpA*	5′-CGGCGATAAAGACATCTTCAC-3′ 5′-GCAACGCGGCCTGGCGGAAG-3′	489	13702525

**Table A3:** The list of the primers (Genomed, Gdynia, Poland) used for the virotyping of the *E. coli* strains.

Gene	Sequences of Primers	PCR Product Size (bp)	Gene Bank ID	Biological (Enzymatic) Function
*astA*	5′-CCATCAACACA-3′ 5′-TCAGGTCGCGAGTG-3′	116	AF143819	Gene encoding heat-stable toxin 1 (ST1)
*Iss*	5′-ATCACATAGGATTCTGCCG-3′ 5′-CAGCGGAGTATAGATGCCA-3′	309	X52665	Gene encoding outer membrane protein of increased serum survival
*irp2*	5′-GGATTCGCTGTTACCGGAC3′ 5′-AACTCCTGATACAGGTGGC-3′	413	L18881	Gene encoding iron binding protein
*iucD*	5′-ACAAAAAGTTCTATCGCTTCC-3′ 5′-CCTGATCCAGATGATGCTC-3′	714	M18968	Gene encoding siderophore’s complex
*tsh*	5′-ACTATTCTCTGCAGGAAGTC-3′ 5′-TTCCGATGTTCTGAA-3′	824	AF218073	Gene encoding thermolabile haemagglutinin
*vat*	5′-TCCTGGGACATAATGGTCAG-3′ 5′- GTGTCAGAACGGAATTGT-3′	981	AY151282	Gene encoding vacuolating autotransporter toxin
*fyuA*	5′-TGATTAACCCCGCGACGGGAA-3′ 5′-CGCAGTAGGCACGATGTTGTA-3′	880	Z38064	Gene belonging to the operon encoding aerobactin siderophore complex at the plasmid ColV-K30
*stx2f*	5′-ATCCTATTCCCGGGAGTTTACG-3′ 5′-GCGTCATCGTATACACAGGAGC-3′	338	912579	Gene encoding Shiga like toxin
*hlyE*	5′-GGTGCAGCAGAAAAAGTTGTAG-3′ 5′-TCTCGCCTGATAGTGTTTGGTA-3′	569	13701006	Gene encoding haemolysin
*fimC*	5′-GTTGATCAAACCGTTCAG-3′ 5′-AATAACGCGCCTGGAACG-3′	424	948843	Gene encoding fimbriae type I
*eae*	5′-TCAATGCAGTTCCGTTATCAGTT-3′ 5′-GTAAAGTCCGTTACCCCAACCTG-3′	482	AF022236	Gene encoding intimin
*bfp*	5′-GGAAGTCAAATTCATGGGGGTAT-3′ 5′-GGAATCAGACGCAGACTGGTAGT 3′	246	KJ020697	Gene encoding bundle forming pilli
*cvi/* *cva*	5′-TGGTAGAATGTGCCAGAGCAAG-3′ 5′-GAGCTGTTTGTAGCGAAGCC-3′	1181	AJ223631	Gene encoding colicin at the ColV plasmid
*papC*	5′-GTGGCAGTATGAGTAATGACCGTTA-3′ 5′-ATATCCTTTCTGCAGGGATGCAATA-3′	200	X61239	Gene encoding P fimbriae subunit
*kpsMTII*	5′-GCGCATTTGCTGATACTGTTG-3′ 5′-CATCCAGACGATAAGCATGAGCA-3′	272	X53819	Gene encoding extracellular capsules type II biosynthesis proteins
*kpsMT (K1)*	5′-TAGCAAACGTTCTATTGGTGC-3′ 5′-CATCCAGACGATAAGCATGAGCA-3′	153	M57382	Gene encoding capsules antigen K1
*kpsMTIII*	5′-TCCTCTTGCTACTATTCCCCCT -3′ 5′-AGGCGTATCCATCCCTCCTAAC-3′	392	AF007777	Gene encoding extracellular capsules type III biosynthesis proteins
*rfc*	5′-ATCCATCAGGAGGGGACTGGA-3′ 5′-AACCATACCAACCAATGCGAG -3′	788	U39042	Gene encoding O-side-chain antigen of LPS
*traT*	*5′-* GGTGTGGTGCGATGAGCACAG-3′ 5′-CACGGTTCAGCCATCCCTGAG-3′	290	J01769	Gene encoding serum resistance and pathogenicity-related protein

**Table A4:** Oligonucleotides used in the MLST method, their size and Gene Bank ID.

Gene	Primer’s Sequences	PCR Product Size (bp)	Biological (Enzymatic) Function
*adk*	5’-ATTCTGCTTGGCGCTCCGGG-3’ 5’-CCGTCAACTTTCGCGTATTT-3’	583	adenylate kinase
*fumC*	5’-TCACAGGTCGCCAGCGCTTC-3’ 5’-GTACGCAGCGAAAAAGATTC-3’	806	fumarate hydratase
*gyrB*	5’-TCGGCGACACGGATGACGGC-3’ 5’-GTCCATGTAGGCGTTCAGGG-3’	911	DNA gyrase
*icd*	5’-ATGGAAAGTAAAGTAGTTGTTCCGGCACA-3’ 5’-GGACGCAGCAGGATCTGTT-3’	878	isocitrate dehydrogenase
*mdh*	5’-AGCGCGTTCTGTTCAAATGC-3’ 5’-CAGGTTCAGAACTCTCTCTGT-3’	932	malate dehydrogenase
*purA*	5’-CGCGCTGATGAAAGAGATGA-3’ 5’-CATACGGTAAGCCACGCAGA-3’	816	adenylosuccinate synthetase
*recA*	5’-ACCTTTGTAGCTGTACCACG-3’ 5’-TCGTCGAAATCTACGGACCGGA-3’	780	ATP/GTP binding motif
